# Immunomodulatory and Regenerative Effects of Mesenchymal Stem Cells and Extracellular Vesicles: Therapeutic Outlook for Inflammatory and Degenerative Diseases

**DOI:** 10.3389/fimmu.2020.591065

**Published:** 2021-02-05

**Authors:** Sylwia Dabrowska, Anna Andrzejewska, Miroslaw Janowski, Barbara Lukomska

**Affiliations:** ^1^ NeuroRepair Department, Mossakowski Medical Research Centre, PAS, Warsaw, Poland; ^2^ University of Maryland School of Medicine, Baltimore, MD, United States; ^3^ Center for Advanced Imaging Research, Department of Diagnostic Radiology and Nuclear Medicine, University of Maryland School of Medicine, Baltimore, MD, United States

**Keywords:** extracellular vesicles, mesenchymal stem cells, immunomodulation, protection, regeneration

## Abstract

Mesenchymal stem cells (MSCs) are non-hematopoietic, multipotent stem cells derived from mesoderm, which can be easily isolated from many sources such as bone marrow, umbilical cord or adipose tissue. MSCs provide support for hematopoietic stem cells and have an ability to differentiate into multiple cell lines. Moreover, they have proangiogenic, protective and immunomodulatory properties. MSCs have the capacity to modulate both innate and adaptive immune responses, which accompany many diseases, by inhibiting pro-inflammatory reactions and stimulating anti-inflammatory activity. Recent findings revealed that the positive effect of MSCs is at least partly associated with the production of extracellular vesicles (EVs). EVs are small membrane structures, containing proteins, lipids and nuclei acids, which take part in intra-cellular communication. Many studies indicate that EVs contain protective and pro-regenerative properties and can modulate an immune response that is activated in various diseases such as CNS diseases, myocardial infarction, liver injury, lung diseases, ulcerative colitis or kidney injury. Thus, EVs have similar functions as their cells of origin and since they do not carry the risk of cell transplantation, such as tumor formation or small vessel blockage, they can be considered a potential therapeutic tool for cell-free therapy.

**Graphical Abstract:**
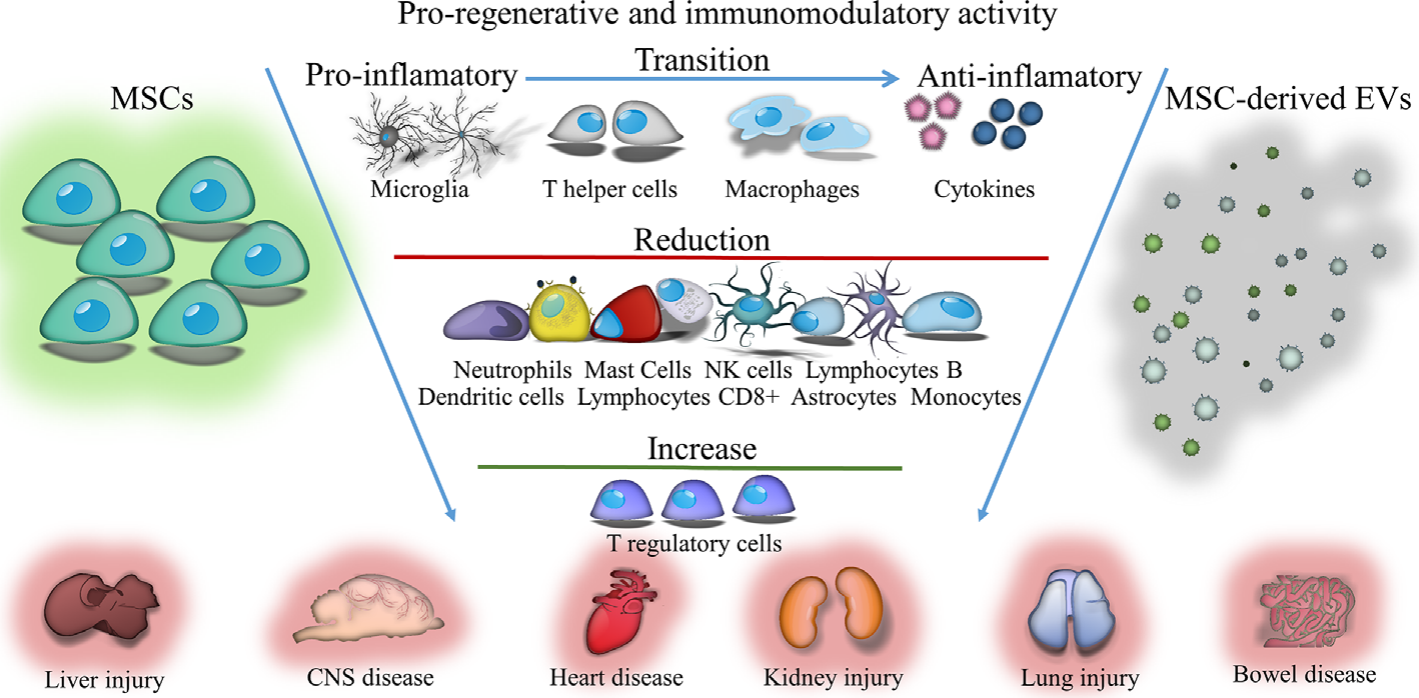
Immunomodulatory and regenerative effects of mesenchymal stem cells (MSCs) or extracellular vesicles (EVs) derived from them in inflammatory and degenerative diseases.

## Introduction

Immunomodulation is increasingly being considered as a therapeutic alternative for an array of incurable diseases. It frequently is achieved by blocking harmful effects of the immune response while also promoting beneficial interactions of the immune system with disordered tissues. There is growing evidence that mesenchymal stem cell (MSC) transplantation has a positive effect by stimulating repair processes after injury and modulating the recipient’s immune response. This cross-talk between donor MSCs and recipient cells seems extremely important for normalization of homeostasis of the body and for survival and preservation of functions after cell damage. Hence, the development of “immunomodulatory strategies,” used to control the functions of individual immune cell populations at various stages of disease, seems to be a promising therapeutic approach. The transplantation of MSCs, which has strong immunomodulatory properties, may be one of them. Due to the strong paracrine properties, transplanted MSCs release a number of trophic factors into the host environment that are involved in reducing inflammation, apoptosis and fibrosis of damaged tissues. Studies conducted in recent years have shown that the therapeutic effects of MSCs can be also conveyed through extracellular vesicles (EVs). It seems that EVs released from MSCs may perform the same functions as the cells from which they originate. Previous studies revealed that the content of EVs transferred to surrounding cells can change their fate and properties. Being natural carriers of bioactive substances, EVs administered under ischemic conditions can reprogram endogenous cells in damaged tissue, modulating their functions and immunosuppressive activity, and limiting the pro-inflammatory response. Therefore, verification of extracellular vesicle effects in preclinical studies compared to MSC transplantation is justified.

### MSCs: Discovery and Sources

MSCs are non-hematopoietic, multipotent stem cells, as evident by their name, which is derived from the middle germ layer - mesoderm. They were first found in the bone marrow by Friedenstein in the late 1960s and described as fibroblast-like cells, adherent to plastic, forming clones, with the ability to self-renew *in vitro* and differentiate into osteoblasts, chondrocytes and adipocytes (1). For many years, MSCs have been called bone marrow stromal stem cells (2, 3). The name “mesenchymal stem cells” was first introduced by Caplan in 1991 (4). Many researchers refer to MSCs as “mesenchymal stromal cells” because of their origin. In 2017, Caplan suggested that the name of MSCs should be changed to “medicinal signaling cells” to emphasize the fact that MSCs migrate to the site of damage or pathology due to disease-related secretion of biologically active regenerative and immunomodulatory factors (5). Bone marrow is one of the most widely used sources of MSCs, in which they constitute 0.001% to 0.01% of mononuclear cells (6–8). MSCs also can be isolated from other sources. Isolation procedure for MSCs from various tissues from adult donors is not very invasive and does not raise ethical and legal objections. Many researchers obtain MSCs from tissues discarded at delivery such as umbilical cord blood (9, 10), Wharton’s jelly umbilical cord (11, 12), placenta (13, 14) and amniotic fluid (15). In recent years, more and more research has been conducted using MSCs isolated from adipose tissue (16, 17). In addition, it is possible to obtain MSCs from other sources, such as skin (18), lungs (19), liver (20) dental pulp (21), endometrium (22), fallopian tube (23), and anterior or posterior cruciate knee ligaments (24).

### Characterization of MSCs

In accordance with the recommendations of the Mesenchymal Stem Cells and Tissue Stem Cells Committee of the International Society for Cell Therapy, in addition to the proliferative and self-renewing characteristics of all stem cells, human MSCs must meet the following criteria: ability to adhere to and grow on plastic under standard laboratory conditions; presence of markers typical for MSCs: CD105, CD73, CD90, in at least 95% of cells and no markers of hematopoietic cells: CD45, CD34, CD14 or CD11b, CD79α, CD19, human leukocyte antigens class II, measured by flow cytometry; ability to differentiate into three types of cells: osteoblasts, adipocytes and chondroblasts under standard *in vitro* conditions. These criteria are typical of human MSCs. MSCs isolated from other animal species must be able to adhere to the surface of plastic and to differentiate into three cell types, but may differ in expression of surface antigen (25).

MSCs grown in culture on a plastic surface have an elongated, fusiform shape, and their morphology resembles fibroblasts. Initially, the MSC population was thought to be more than 98% homogeneous and consist of identical, symmetrical, spindle cells (8). However, later studies have shown that MSC colonies are heterogeneous and consist of smaller spindle cells capable of faster self-renewal and larger cubic-shaped cells or flattened, slower proliferating cells. The Colter group presented a third type of cells, described as very small cells capable of rapid self-renewal, showing the greatest potential for differentiation and considered early cell progenitors (26). In *in vitro* culture, MSCs seeded on a plastic surface reach confluence after 5 to 7 days. After covering the entire area of the flask they begin to grow in several layers, change their shape, flatten and their numerous branches become visible (27). MSCs are capable of 25 to 40 doubling the population, which is 8 to 15 passages, while retaining the ability to differentiate (28). Under laboratory conditions, MSCs grow in three phases: the lag phase lasting 3 to 4 days, the log phase and the stationary phase (26). Studies have shown that phase change is associated with different expression of genes, including Dickkopf genes, whose highest levels are observed in the phase of rapid growth and Wnt5a genes, which are expressed at the highest level in the stationary phase (29). In addition, MSC cell cycle analysis showed that the majority, as much as 89% of the cells, are in the G0/G1 phase, 6% in phase G (2)/M and 5% in phase S of the cell cycle. These cells retain a normal karyotype (11). *In vivo*, MSCs occupy special places in various body tissues called stem cell niches. The specific microenvironment prevailing in niches and interactions with other cells cause MSCs to remain in dormant and are activated only under the influence of a damaging, pathogenic or aging factor. The hypothesis of the existence of a niche of stem cells in the bone marrow was proposed by Schofield in the 1970s (30). The Wnt signaling pathway is probably involved in the mechanism that allows cells to remain dormant or begin the differentiation process (31).

In addition to markers used in the classification of MSCs, such as CD105 (SH2), CD73 (SH3 and SH4) and CD90 (Thy-1), MSCs derived from bone marrow show the presence of other antigens, including: CD10, CD13, CD146, CD271, CD44, Stro-1, stage-specific embryonic antigen-4, neurotic ganglioside, 3G5, stage-specific embryonic antigen 3, stem cell antigen 1, Stro-4, mesenchymal stromal cell antigen-1, PODXL, Sox1, and TM4SF1 (32). MSCs do not have proteins typical of hematopoietic and endothelial cells: CD11b, CD14, CD31, CD33, CD34, CD133, and CD45 (8). In addition, MSCs possess messenger RNA (mRNA) for cell adhesion molecules such as CD106 (vascular cell adhesion protein 1), CD166 (a molecule activating cell leukocyte adhesion), CD29 (beta 1 integrin), CD49a, b, c, e, f (integrin alpha 1, 2, 3, 4, and 6), integrin alpha 11, CD51 (alpha V integrin), CD54 (intercellular adhesion molecule 1), E-cadherin, CD56 (neuron adhesion molecules) (33). Studies have shown that on the surface of MSCs derived from bone marrow, receptors for chemokines from the CC group are present, including CCR1, CCR3, CCR7, CCR9, CCR10, and from the CXC group, including such as CXCR3, CXCR4, CXCR5, and CXCR6 (33, 34).

An important feature of MSCs is their low immunogenicity. They show low expression of major histocompatibility complex (MHC) class I proteins, and they do not express co-stimulatory molecules. Many *in vitro* studies have shown that MSCs do not induce an immune response from allogeneic lymphocytes (35). *In vivo* experiments confirmed that MSCs do not induce a typical immune response after allogeneic transplantation, which makes it possible to use MSCs from other donors in clinical therapies (36). However, other researchers indicated that MSCs under specific environmental conditions may induce immune responses. Khan and colleagues have shown that MSCs exhibit the function of antigen presenting cells and activate T lymphocytes under the influence of interferon-γ (IFN-γ) (37). Eliopoulos et al. observed that subcutaneous allogeneic MSCs were rejected in unimmunized mice, accompanied by an increase in IFN-γ (38). However, despite the negative results of some research groups, most studies have confirmed the low immunogenicity of MSCs and their survival in the recipient after allogeneic and even xenogeneic transplantation.

### Properties and Mechanism of Action of MSCs

Growth factors, chemokines and cytokines, secreted by damaged cells have the ability to stimulate and mobilize MSCs. Under the influence of mediators such as vascular endothelial growth factor (VEGF), stromal-derived factor 1 (SDF-1), granulocyte colony stimulating factor (G-CSF), granulocyte and macrophage colony-stimulating factor (GM-CSF), erythropoietin, angiopoietin 2, placental growth factor, platelet-derived growth factor (PDGF), stem cell growth factor (SCF), insulin-like growth factor (IGF-1), epidermal growth factor (EGF), hepatocyte growth factor (HGF), and cytokines, including IL-1β, IL-2, IL-3, IL-6, IL-8, and tumor necrosis factor α (TNF-α) and chemokines, including CCL5 and CCL22, MSCs are activated, migrate, and settle in the appropriate tissues (39, 40). Proteins present on the surface of MSCs, such as receptors for CC and CXC chemokines, beta 1 and alpha 4 integrins, and vascular cell adhesion protein 1 and intercellular adhesion molecule 1 integrin ligands are involved in the movement and colonization of MSCs (41). After colonization of the target site in the tissue, MSCs proliferate and differentiate into different cell types. These processes are regulated by the interactions of the Wnt, Notch protein signaling pathways and Hedgehog (31). MSCs can multiply and then transform into specialized cells that make up the tissue and replace dying cells. MSCs have been shown to have the ability to differentiate not only into mesodermal cells: osteoblasts, chondrocytes, adipocytes (8) and myocytes (42, 43), but in endodermal line cells: hepatocytes (44, 45), islet cells (46) or the ectodermal line: neurons (47). In addition, MSCs can associate with other types of cells *via* cell fusion, thus “saving” damaged or dying cells (48).

The action of MSCs can be associated not only with a direct mechanism, through their differentiation and replacement of damaged cells, but primarily with their paracrine properties. MSCs produce a number of substances, such as cytokines, chemokines and growth factors that can protect and regenerate other cells, as well as stimulate their proliferation and differentiation. The most important secreted growth factors include: transforming growth factor β (TGFβ), VEGF, IGF-1, SDF, GM-CSF, G-CSF, HGF, EGF, fibroblast growth factor (FGF), beta nerve growth factor (NGFβ), macrophage colony-stimulating factor, and leukemia inhibitory factor (LIF). In addition, MSCs produce a number of cytokines, including IL 1α, IL-1β, IL-2, IL-6, IL-7, IL-8, IL-10, IL-11, IL-12, IL-14, IL-15, TNFα, and chemokines: CXCL12 (SDF-1), CCL2, CCL5, which are responsible for many functions of MSCs, primarily for their immunomodulatory activity (46–52). They produce extracellular matrix proteins, such as fibronectin, laminin, collagen, and proteoglycans (27).

MSCs can transfer to other cells using tunneling nanotubes not only numerous molecules such as proteins and nucleic acids but also even whole organelles, mainly mitochondria. The Prockop group was the first to indicate that human MSCs co-cultured with a line of lung epithelial cells, devoid of mitochondria, protect cell damage by transferring mitochondria and mitochondrial DNA (53). The transfer of mitochondria through MSCs *via* tunneling nanotubes to other types of cells, such as endothelial cells or cardiomyocytes, has also been described by others (48).

### Biological Functions of MSCs

One of the earliest known functions of MSCs is to provide an adequate microenvironment for hematopoietic stem cells in the bone marrow. MSCs are a mechanical support for hematopoietic cells due to the production of numerous extracellular matrix proteins such as fibronectin, laminin, collagen and proteoglycans (54). In addition, thanks to secreted cytokines, chemokines and growth factors, MSCs regulate hematopoiesis. *In vitro* studies using cell co-culture have shown that MSCs produce growth factors, such as SCF and GM-CSF and interleukins, including IL-6, IL-7, and IL-11, activating hematopoietic cells to proliferate and differentiate towards lymphocytes, monocytes, basophils, eosinophils, erythrocytes, as well as megakaryocytes (55). CXCL12 chemokine produced by mesenchymal cells has been proven to play a key role in growth and maintaining hematopoietic stem cells in bone marrow stem cell niches (56). Studies by Muguruma et al. have shown that MSCs transplanted into mice can differentiate into pericytes, myofibroblasts, stromal cells, osteocytes and endothelial cells, and promote the increase of functionally and phenotypically of immature hematopoietic cells (57).

Participation in the process of angiogenesis is another important task of MSCs. MSCs produce numerous growth factors, cytokines and chemokines that induce endothelial cell proliferation, migration and survival, and activate blood vessel formation and maturation (58). Studies of Kinnaird and Hung groups proved that MSCs activate the proliferation and migration of endothelial cells by secreting the proangiogenic factors VEGF and FGF by inducing angiogenesis (59, 60). The results of the experiments of Wu et al. showed that transplanted mesenchymal bone marrow cells are involved in wound healing through the release of VEGF and angiopoietin 1 (61).

Another function of MSCs is their neuroprotective effect on damaged or dying nerve cells, through the production of various growth factors and cytokines. Studies carried out by many authors have proven that mediators secreted by MSCs, such as brain-derived neurotrophic factor (BDNF), gliogenic neurotrophic factor (GDNF), neurotrophin-3, NGF, and VEGF, have neuroprotective effects on nerve cells, causing them to survive and activate growth (62). Johnson et al. demonstrated that the protective effect of MSCs on nerve cells is associated with the production of 11 factors: BDNF, IFN-γ, IL-6, IL-11, LIF, NGF, PDGF-AA, PDGFAB/BB, SCF, melatons, and thrombospondin 1 (63). In addition, MSCs through the release of factors, including those such as TGF-β, VEGF, or IL-6, act neuroprotectively on scraps of nerve tissue damaged by oxygen and glucose deprivation (49).

Mesenchymal cells show protective properties not only against neuronal cells but also against other types of cells. Studies by Preda et al. have shown that transplanted MSCs perform cardioprotective functions, protecting myocardial cells against ischemia-reperfusion injury (64). Other experiments indicated that MSCs have protective effects on cells in a model of acute ischemia-induced kidney damage due to paracrine activity (65). Experiments using co-culture of pancreatic islets with mesenchymal cells derived from rat bone marrow proved that cytoprotective and anti-apoptotic properties of MSCs are associated with IL-6 and TGF-β secretion and expression of anti-apoptotic genes, such as Mapkapk2, Tnip1 and Bcl3 (66).

As a result of their ability to self-renew and differentiate into different cell lines, MSCs perform regenerative functions by replacing dying cells in various tissues. According to standard protocols, MSCs *in vitro* under the influence of dexamethasone, glycerophosphate and ascorbic acid differentiate into osteoblasts showing the presence of mineral aggregates and increased alkaline phosphatase activity. Research by Pittenger and other authors has shown that factors such as bone morphogenetic proteins and growth factors such as TGF, IGF-1, BDNF, and FGF-2 are involved in the osteogenesis process (8, 27). In turn, under the influence of the medium used for culture enriched with insulin-transferrin-selenium, linoleic acid, selenic acid, pyruvate, ascorbate 2-phosphate, dexamethasone and TGF-β, MSCs cells differentiate into collagen-containing chondrocytes (8, 67). MSCs which grow in the medium with the addition of dexamethasone, indomethacin, isobutyl, methyl and insulin may differentiate into adipocytes, and after three weeks exhibit the presence of fat drops and the expression of genes specific for these cells, including receptors activated by γ-peroxisome proliferators, adipocyte proteins 2 and lipoprotein lipase (8, 68).

Additionally, studies conducted by Xu et al. have shown that MSCs derived from rat bone marrow can differentiate under the influence of 5-azacytidine into cardiomyocytes, expressing genes specific for myocardial cells, including myosin β heavy chain, α cardiac actin and desmin (43). The experiments of Alimperti et al. have shown that MSCs exposed to cadherin-11 and TGF-β1 transform into functionally active smooth muscle cells (42). Under specific *in vitro* culture conditions, mature hepatocytes may be generated from human bone marrow stem cells (hBM-MSCs). In the first stage, the cells differentiate under the influence of EGF-enriched medium, basic fibroblast growth factor (bFGF) and nicotinamide, and then ripen with oncostatin M, dexamethasone and insulin-transferrin-selenium (44, 45). Studies performed by Bai et al. have shown the possibility of differentiation of MSCs into pancreatic islet cells, expressing Pdx1, insulin, and C-peptide (46). A few literature reports indicate that mesenchymal cells have the ability to differentiate into neuronal cells. Experiments confirmed the possibility of *in vitro* transformation of MSCs into mature neurons under the influence of growth factors such as bFGF, EGF, HGF, NT3, GDNF, and BDNF (47, 67). However, under the influence of β-mercaptoethanol, retinoic acid, bFGF, PDGF, forskolin, and heregulin, MSCs differentiated into Schwann cells (69).

### Immunomodulatory Functions of MSCs

Very important functions of MSCs are their immunomodulatory properties. MSCs have the ability to regulate mechanisms of both innate, as well as adaptive immune response, through the modulation of cellular responses and the secretion of inflammatory mediators. The immunomodulation process takes place in many stages and includes: recognition of the inflammatory response and migration of MSCs to the site of injury, “licensing” or activation of MSCs, if necessary, induction of pathogen removal, and modulation of inflammation (40).

MSCs grown in medium supplemented with human serum activate the complement system, which results in their damage (70). MSCs isolated from bone marrow have been shown to have receptors on their surface for complement components such as C3a and C5a. They are chemotactic factors for MSCs and increase their resistance to oxidative stress, as well as the activation of signal pathways responsible for proliferation and anti-apoptotic mechanisms (71). The expression of protectin (CD59) also has been identified on the surface of MSCs and has been shown to secrete factor H. Both molecules inhibit the complement system, which may partially protect MSCs from damage by complement components. They also have been shown to block complement-induced proliferation of peripheral blood mononuclear cells, which is one of the mechanisms of their anti-inflammatory activity (70, 72).

Numerous studies have proven that MSCs can regulate the activity of macrophages and microglia cells. MSCs, as well as the supernatant from their culture, stimulate macrophage migration *in vitro*, and increase their *in vivo* mobilization to the healing tissue area, accelerating its renewal process (73). Interacting with macrophages, MSCs promote their polarization from the M1 pro-inflammatory phenotype to M2 anti-inflammatory phenotype cells, reduce the production of pro-inflammatory cytokines such as TNF-α, IL-1, IL-6, IL12p70, IFN-γ, and increase secretion of anti-inflammatory cytokines, including IL-10, IL-12p40 (74, 75). Francois and co-workers demonstrated that TNF-α and IFN-γ-activated MSCs, derived from the adult volunteers, suppress T cell proliferation *in vitro.* This effect was linked to IFN-mediated indoleamine 2,3-dioxygenase (IDO) up-regulation (74). Conversion of monocytes into M2 immunosuppressive macrophages are likely to amplify the overall suppressive effect of MSCs observed *in vivo*. These findings have been confirmed by Monguio-Tortejada and coworkers who showed that human AT-MSCs and UC-MSCs promote polarization of monocytes toward regulatory M2 phenotype (76). The authors demonstrated that MSCs upregulate the CD39 and CD73 expression on monocytes *in vitro.* The CD39 and CD73 regulate the purinergic signaling by the hydrolysis of ATP/ADP to AMP and to adenosine, respectively. This induces the shifts from the pro-inflammatory milieu induced by extracellular ATP into the anti-inflammatory environment regulated by Ado (76). Upregulation of these ectonucleotidases on endogenous monocytes after MSC transplantation would have the advantage of the host monocyte migration and their delivery to the site of inflammation, shifting pro-inflammatory milieu to anti-inflammatory environment. In addition, the local treatment with porcine AT-MSCs using an engineered bioactive graft was shown to promote the *in vivo* CD73 expression on the host monocytes in a swine model of myocardial infarction (76). *In vitro* studies performed by Giunti et al. revealed that MSCs reduce microglia activation by inflammatory factors. The presence of mouse BM-MSCs significantly increase microglial expression and release of molecules associated with a neuroprotective cell phenotype such as CX3CR1, nuclear receptor 4 family, CD200 receptor, and insulin growth factor 1 (77).

MSCs also can modulate neutrophil activity. Experiments have shown that interleukin-6 produced by MSCs activates signal transducer and activator of transcription 3 factors, which results in increased survival of neutrophils (78), reduces the expression of genes characteristic for cells entering the path of programmed cell death, such as Bax and MCL-1, and also limits the oxygen explosion. On the other hand, IL-6 reduces the production of reactive oxygen species by neutrophils which weakens the adverse effects of these cells (79). In addition, mesenchymal cells secrete IDO, which inhibits the production of stored α-denfensin in secretory granules of neutrophils with pro-inflammatory properties (80). In turn, prostaglandin-2 release by MSCs stimulates macrophages and microglia cells for the production of IL-10, which limits the influx of neutrophils to damaged tissue. Secreted IL-10 also acts on endothelial cells, causing a reduction in E-selectin expression and inhibiting neutrophil migration to the site of injury (78).

Studies also have shown that the co-culture of MSCs with mast cells reduces mast cell degranulation, secretion of pro-inflammatory cytokines such as TNF-α as well as their migration towards the chemotactic factor. It has been proven that MSCs administered to the inflamed area reduce allergic reaction-induced degranulation and infiltration of this area by mast cells *in vivo* (81). In the presence of mast cells, MSCs enhance the expression of induced cyclooxygenase-2 (COX-2) and increase the production of PGE-2, which binds to the EP4 receptor on the surface of mast cells. This leads to their deactivation (82). MSCs have H1, H2, and H4 receptors on their surface that bind histamine - the main mediator secreted by mast cells during an allergic reaction. Their activation leads to an increase in IL-6 production by MSCs, which may affect, among others, regulation of neutrophil survival and migration (83).

Studies to date have shown that MSCs have immunosuppressive effects on natural killer (NK) cells. MSCs inhibit the proliferation of naturally cytotoxic cells, by producing IDO and PGE-2, showing synergistic activity (84). Other experiments indicate that the factor responsible for inhibiting NK cell proliferation is released by MSCs TGF-β (85). In addition, MSCs limit IL-2 and IL-15 production, which results in reduced NK cell proliferation and IFN-γ production (35). MSCs also have the ability to reduce the expression of NK cell activating receptors, including NKp30, NKp44 and NKG2D, which contribute to a decrease in the cytotoxic activity of these cells and to limit the secretion of pro-inflammatory cytokines (84). Activated NK cells attack MSCs leading to their lysis due to the low level of MHC I expression on their surface. Exposure to interferon γ induces an increase in MHC I expression in MSCs which blocks cell cytotoxicity (86).

**Figure 1:**
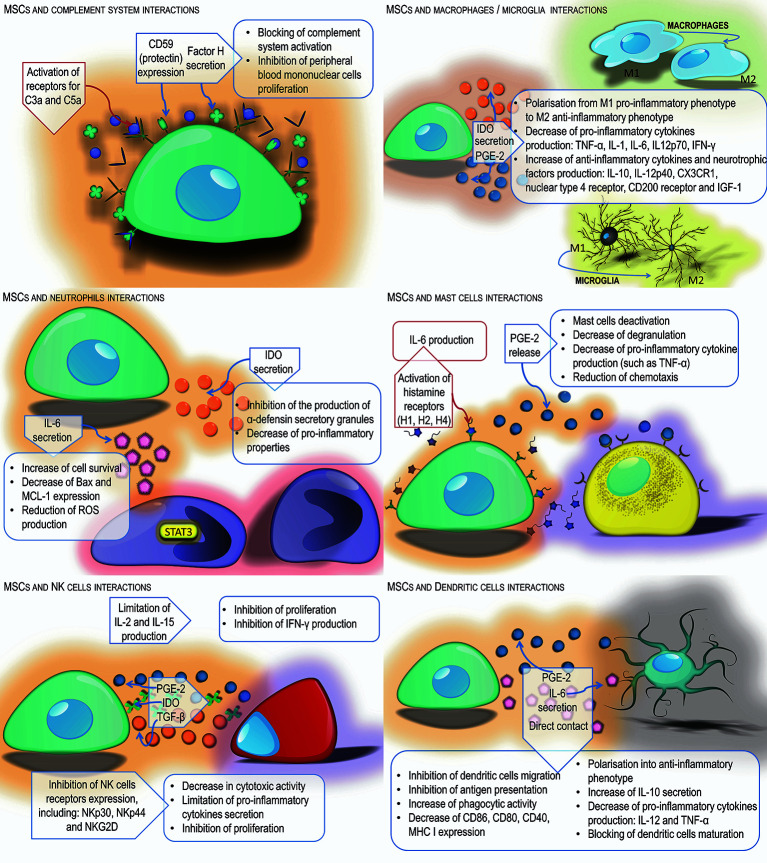
Interactions of MSCs with innate immune cells. MSCs exert immunomodulatory effects on neutrophils, NK cells, macrophages/microglia, mast cells, and dendritic cells through secretion of various soluble factors or direct cell-cell interaction.

MSCs interact with dendritic cells (DCs), limiting their functions, including migration capacity, maturation, and antigen presentation. Numerous experiments have confirmed that MSCs reduce the expression of markers of mature DCs including MHC class II molecules, CD40, CD80 and CD86 and modulate the expression of markers of “DCs deposition” in lymph nodes, including CCR7 chemokines (87). Studies by Li et al. have shown that regulation of DC maturation can be mediated by cytokines produced by MSCs, such as IL-6, or by direct contact using the Notch signaling pathway (88). MSCs also can affect the transformation of immature DCs into mature forms. They block DC maturation by secreting PGE-2 (89). MSCs have the ability to change the phenotype of DCs to anti-inflammatory. DCs under the influence of MSCs increase the secretion of anti-inflammatory cytokines, including IL-10, decrease the production of pro-inflammatory cytokines such as IL-12 and TNF-α and increase their phagocytic activity. In addition, experiments by Zhang et al. have shown that phenotypically altered DCs inhibit hypersensitivity reactions *in vivo* (90, 91), and are not able to activate T helper (Th) cells (92); instead they induce the formation of antigen-specific T regulatory (Treg) cells (88) (**Figure 1**).

MSCs can modulate the action of secondary immune response cells. One of the tasks of MSCs is maintaining a proper balance between Th1 and Th2 phenotype CD4 T cells. Numerous *in vitro* and *in vivo* studies have shown that MSCs activate the change of CD4 T cells with a Th1 inflammatory phenotype, secreting IL-1α, IL-1β, IFN-γ, and TNF-α into Th2 anti-inflammatory phenotype cells producing IL-3, IL -4, IL-5, IL-10, and IL-13 (93, 94). Conversely, in allergic diseases, MSCs reduce the production of Th2 dependent cytokines and increase the secretion of Th-1–dependent cytokines, which provides an appropriate balance and protects in allergic respiratory diseases (95). In addition, MSCs, by secreting indoleamine 2,3-dioxygenase and prostaglandin E-2, inhibit the differentiation of IL-17–producing Th17 lymphocytes, in favor of the formation of Th2 lymphocytes and Treg lymphocytes (93, 96). The TGF-β produced by MSCs plays an important role in maintaining an appropriate balance between the Th1, Th2 and Th17 helper lymphocytes and regulatory lymphocytes (97).

Another cells affected by MSCs are CD8 T cells. By suppressing Th1 and Th17 lymphocytes necessary for the activation of CD8 T cells, MSCs contribute to the suppression of these cells (78). Experiments have shown that MSCs can also directly prevent the cytotoxic activity of CD8 lymphocytes, mainly by blocking their proliferation rather than by inhibiting the cytotoxic effect (98).

As a result of direct contact with MSCs or through factors produced by them, such as IDO, PGE-2, and TGF-β, the formation of antigen-specific regulatory T cells occurs. *In vitro* studies have shown that Treg cells affect Th lymphocytes with a pro-inflammatory phenotype causing inhibition of their proliferation (99). In contrast, IL-4 and IL-13 produced by Th2 lymphocytes stimulate MSCs to produce TGF-β, which is an important factor that stimulates regulatory T cells (97). In addition to the aforementioned factors, nitric oxide, galectin-1, and semaphorin-3A produced by MSCs stimulate lymphocyte differentiation toward Treg (48). Treg activation is also the main mechanism induced by MSCs enabling the acceptance and survival of allogeneic transplants (100), as well as protection against some autoimmune and allergic reactions (40).

**Figure 2:**
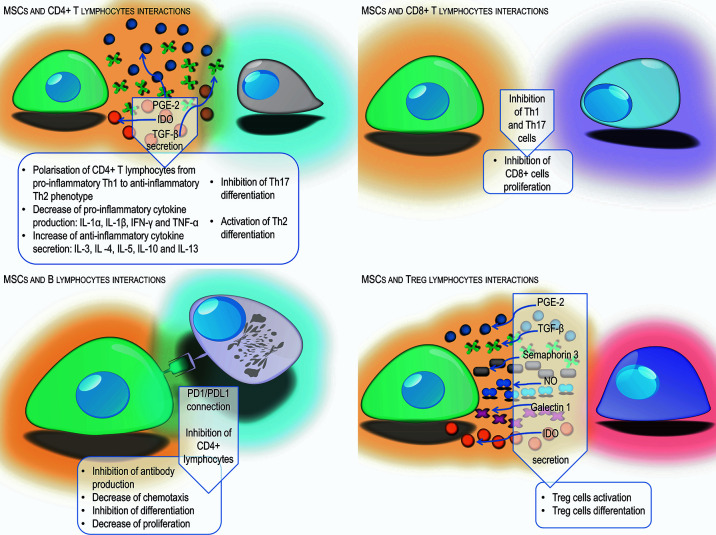
Schematic representation of different mechanisms used by MSCs to suppress the adaptive immune response from T and B lymphocytes by cell-cell interaction and secretion of different factors.

Mesenchymal cells also affect B lymphocytes, retaining them in the G0/G1 phase, limiting their chemotactic activity (101). Studies have shown that MSCs have the ability to reduce the activity, differentiation and proliferation of B lymphocytes *via* the PD-1 signaling pathway (102). In addition, MSCs act as a suppressor on CD4 T cells, resulting in reduced production of the CD40L co-stimulatory molecule and inability to activate B cells (78). The experiments of Corcione et al. have shown that MSCs have the ability to inhibit the production of antibodies (101). Other researchers have described the possibility of MSCs stimulating proliferation and B cell differentiation (103) (**Figure 2**).

### Extracellular Vesicles

In addition to direct actions, MSCs can modify the functions of the microenvironment through the release of extracellular vesicles. MSCs transmit numerous proteins, including cytokines, chemokines and growth factors, membrane receptors, lipids and various types of nucleic acids through EVs, thus protecting and regenerating the damaged cells and mitigating the immune response. EVs are thought to play a key role in intercellular communication. The Nobel Prize in Physiology and Medicine in 2013 was awarded for the discovery of transport between cells in vesicles (104).

### Types of EVs

EVs are membrane structures secreted by cells, including MSCs. They constitute a heterogeneous population consisting of several types of EVs differing in origin, size, shape and content. According to the classic nomenclature, we can distinguish three main types: exosomes, microvesicles and apoptotic bodies (105). Exosomes are currently the most thoroughly described type of EVs: 30 nm to 120 nm in size, spherical in shape and with a two-layer lipid film. They constitute a homogeneous population of EVs, and their optical density is from 1.13 to 1.19 g/cm^3^. Exosomes are formed by the convexity of endosomal membranes in late endosomes, resulting in the formation of multi-alveolar bodies, which by connecting with the cytoplasmic membrane, cells release their content to the outside in the form of exosomes (106). This type of EV is characterized by the presence of many proteins, including annexin, tetraspanin (CD63, CD81, CD9), heat shock proteins, clarins, caveolin, exosome specific proteins such as Alix and Tsg101, and proteins typical of cells from which they come (107–109). In addition, exosomes contain characteristic lipids, including: fragments of lipid raft—GM1 ganglioside, cholesterol, ceramides, sphingomyelin, and phosphatidylserine, as well as nucleic acids: mRNA and microRNA (miRNA) (110, 111).

The second type of EVs is microvesicles called ectosomes or budding vesicles. They range in size from 100 nm to 1 μm, are a heterogeneous population of irregular shape, and their optical density is not well defined. Microvesicles arise directly from the cell’s cytoplasmic membrane, by budding from the membrane surface, and then separating the resulting structures, similar to the detachment that occurs during cytokinesis (112). Microvesicles do not contain proteins involved in the process of endocytosis, but show the presence of integrins, flotillins, selectins, metalloproteinases, CD40 proteins, and some of them have proteins typical of so-called exosomes: tetraspanin. In addition, they are rich in lipids, have large amounts of phosphatidylserine and cholesterol, as well as sphingomyelin and ceramides, and also accumulate nucleic acids: mRNA and miRNA (105, 110).

The third type of EVs is apoptotic bodies also called apoptotic vesicles. They range in size from 50 nm to 4 µl, most of them over 1 µm, and they are characterized by irregular shape (113). The optical density of apoptotic bodies ranges from 1.16 to 1.28 g/cm^3^; they are formed from dying cells that are in the late stage of the process of apoptosis. Inside they contain histones, fragments of organelles and cell membranes, nucleic acids: mRNA, miRNA and DNA, have a high concentration of phosphatidylserine, and their membranes are permeable (112).

### Composition of EVs

EVs contain numerous compounds in their interior, including: proteins, lipids and nucleic acids. The secretome of EVs released by MSCs are not constant due to the heterogeneity of the cell population, isolation methods, different cell passages or sources from which they are isolated (114). Proteomic analysis of EVs isolated from MSCs from various sources allowed the identification of their proteins’ characteristics (115). Proteins transported with EVs are involved in cell-to-cell signaling, cell adhesion, angiogenesis, apoptosis, and immune responses. Previous studies have shown that EVs contain tetraspanins and MSC-typical proteins, surface receptors and signaling molecules involved in self-renewal and cell differentiation (116). In addition, EVs accumulate RAB proteins that regulate the transport and attachment of EVs to cells (117). Various types of biologically active lipids are located in EVs. EV membranes originate from various sources, are rich in lysobiphosphatidic acid molecules that regulate the microvesicles detachment process, as well as in lipid raft lipids, including cholesterol, ceramides, sphingolipids and glycophospholipids (118). In addition, EVs contain lipid mediators, such as prostaglandins, the enzymes responsible for EV formation, including phospholipases A2, C and D and free fatty acids e.g., arachidonic acid (119). RNA molecules are another important component of EVs from various cell types. The most common RNA types present in all types of EVs include mRNA and miRNA (120). A large amount of transporting RNA was observed in EVs derived from MSCs. In addition, ribosomal RNA is present in EVs, varying in number depending on the cell type (121). Recent research also indicates the content of small, non-coding RNA molecules in EVs - piwi-interacting RNA (122). Moreover, many studies have shown that miRNA is the type of RNA involved in MSC-exosome-mediated functional activity (123). It was reported that the transfer of miR-125a and miR-30b induce the tube formation of human umbilical vein endothelial cells, promoting the process of angiogenesis (124, 125). On the other hand, MSC-exosomes contain miR-100 which targets to VEGF in breast cancer cells and determines the anti-angiogenic effect in a tumor microenvironment (126). Other authors proved that exosomal miR-181c and miR-146a have immunomodulatory properties by a downregulation of the Toll-like receptor-4 signaling pathway (127) and an enhancement of M2 macrophages polarization (128). Moreover, miR-233 is responsible for an anti-apoptotic effect of MSC-exosomes and the cardioprotection in sepsis (129). MSC-exosomes contains also miRNA such as: miRNA-181-5p (130) and miR-122 (131) revealing an anti-fibrotic effect in liver fibrosis. Recently it was shown that exosomal miR-124 promote cell proliferation and liver regeneration *via* inhibiting Foxg1 after partial hepatectomy in rats (132).

### Immunomodulatory Functions of EVs Derived From MSCs *In Vitro*

The immunomodulatory properties of MSCs, which have been the subject of many *in vitro* studies, appear to be associated with extracellular vesicles secreted by the cells. It has been shown that EVs released from MSCs have the ability to inhibit the proliferation and differentiation of B lymphocytes, as well as their action is dose-dependent (133). Similarly, dose-dependent inhibition of IgM, IgG, and IgA production by extracellular vesicles derived from mesenchymal cells has been demonstrated. Mokarizadeh et al. observed that EVs produced by murine BM-MSCs have the ability to inhibit the proliferation of syngeneic and allogeneic T lymphocytes, induce apoptosis of activated T lymphocytes, increase in the number of regulatory T lymphocytes and enhance the secretion of anti-inflammatory cytokines: IL-10 and TGF-β1 (134).

Other authors have shown that galectin-1 and programmed death receptor ligand (PD-L1) were present not only on the surface of MSCs but also on EVs derived from MSCs (135). Galectin-1, which is endogenous leptin, has been reported to induce apoptosis of activated T lymphocytes and stimulate maturation of regulatory T lymphocytes (136, 137). In contrast, PD-L1, which is a ligand of the PD-1 receptor, induces the proliferation and activity of regulatory T cells. In addition, EVs derived from mesenchymal stem cells contained TGF-β, which also activates regulatory T cell formation (138).

### Protective/Pro-regenerative and Immunomodulatory Properties of EVs Derived From MSCs *In Vivo*

EVs derived from MSCs (MSC-EVs) have shown protective, regenerative and immunomodulatory properties in animal models *in vivo*. The therapeutic potential of EVs has been pursued in several disease settings. The most commonly studied models of organ injury for MSC-EV therapy are myocardial ischemia and ischemic stroke.

Coronary artery diseases lead to blockage of blood flow followed by ischemia and reperfusion injury. Exosomes obtained from MSCs have shown a protective effect on myocardial cells damaged as a result of acute myocardial infarction. Intravenous infusion of MSC conditioned media before reperfusion reduced infarct size in mouse and pig models of myocardial/reperfusion (MI/R) injury (139, 140). Infarct size reduction in MI/R injury is attained by enhanced cardiomyocyte viability. EVs derived from human MSCs conditioned media reduce oxidative stress and activate pro-survival signaling in cardiac cells preventing adverse cardiac remodeling after MI/R damage. Moreover, EV-treated animals exhibited lower white blood cells counts in the peripheral blood and reduced neutrophil and monocyte influx into the hearts compared to control animals after MI/R injury (141). Administration of MSC-EVs was shown to inhibit apoptosis in cardiomyocytes in a mouse model of acute myocardial infarction (142). Similarly, Sun et al. have shown that MSC-EVs reduced cardiomyocyte apoptosis in an experimental model of dilated cardiomyopathy (143). The authors revealed that intravenous injection of MSC-EVs regulates the balance between two subsets of macrophages observed in the hearts of mice subjected to dilated cardiomyopathy. MSC-EVs decreased the number of pro-inflammatory M1-like macrophages while increasing the number of anti-inflammatory M2-like macrophages. The observed switch of macrophages to an anti-inflammatory state changed the cytokine profiles reducing inflammatory cytokines such as IL-1, IL-6, and TNF-α in serum of dilated cardiomyopathy mice after MSC exosome treatment (143). In a recent study, performed by Wei et al. administration of MSC-EVs carrying miR-181a reported to decrease the expression of pro-inflammatory cytokines such as IL-6 and TNF-α while enhanced the expression of anti-inflammatory IL-10 in mouse heart tissues after MI/R insult (144). Moreover, it was shown that the number of T regulatory cells detected locally was much higher in MSC-EVs or miR-181a overexpressed EV treated mice than the control group subjected to acute myocardial infarction. Additionally, MSC-EVs enriched with miR-19a and miR-221 injected into the border of an ischemic heart region reduced apoptosis downregulating Sema3A and activator of transcription 3 genes regulated cell death (129, 145). Intra-myocardial infusion of MSC-EVs improved cardiac function preserving cardiac systolic and diastolic performance in MI/R rats and mice assessed by echocardiography (146–148). EVs induce neovascularization in ischemic heart disease associated with proliferation and migration of endothelial cells. MSC-EVs treatment revealed significantly higher density of capillaries in infarcted hearts than control animals one month after MI/R injury (146, 147). It was shown that MSC-EVs carry high levels of proangiogenic factors i.e., extracellular matrix metalloproteinase inducer (EMMPRIN) and thus may promote angiogenesis (149). The studies revealed that MSC-EVs contain also such pro-angiogenic factors as: PDGF-D, EGF, FGF, VEGF and SCF (150). Moreover, it was revealed that the main mechanisms responsible for the enhancement of angiogenesis by MSC-EVs are activation of NFκB signaling pathway, transfer of STAT1 and induction of Wnt4/β-catenin signaling pathway (150–152).

EV delivery also offers neuroprotective, neurorestorative, and immunomodulatory effects in neurological diseases. MSC secretome therapy significantly reduced neuronal loss and apoptosis caused by traumatic brain injury (TBI) in rats (153). EV treatment increased the number of newly generated neuroblasts and mature neurons in dentate gyrus and ischemic zones of the brains as well as reduced inflammation in TBI rats (154). MSC-derived EVs from different sources selectively promote neurite growth and remodeling (155–157). It also promotes cerebral endothelial proliferation and new capillary network arrangement assisting in the reconstruction of damaged CNS tissues. Enhanced neurogenesis and angiogenesis observed in MSC-EV recipients positively correlated with spatial learning and memory impaired in mice and rats subjected to stroke (158–160). Moreover, Kim et al. demonstrated that intravenously infusion of EVs after induction of TBI in mice diminished the levels of pro-inflammatory cytokine IL-1β in brain tissue (159). Systemically injected MSC-EVs after ischemic stroke or TBI also improve functional outcome. Delivery of MSC-EVs reduces post-ischemic motor coordination deficits (154, 161). This functional recovery is associated with axonal sprouting, fiber integrity, oligodendrogenesis and re-myelination in experimental models of stroke (162, 163). Furthermore, MSC-EV treatment reduces brain inflammation after brain injury in rats, decreasing microgliosis and preventing reactive astrogliosis (162). In another very recent study, intra-arterial transplantation of EVs from human BM-MSCs led to decrease of pro-inflammatory cytokines: IL-1α, IL-1β, IL-6, TGF-β2, and chemokines: CXCL1, MIP-1α, MIP-3α, and MCP-1 in the striatum of focal ischemic brain injured rats (164). Additionally, it was reported that MSC-EVs have neuroprotective effect in a preclinical model of fetal hypoxic-ischemic brain injury. The study revealed that MSC-EVs improved the structural and functional outcome by preventing hypomyelination, decreasing the number and duration of seizures, and by protecting baroreceptor reflex sensitivity (165). MSC-EVs could facilitate spinal cord injury healing. EVs isolated from human umbilical cord MSCs injected systemically into mice model of spinal cord injury improved functional recovery in transplant recipients (166). Similarly, Lu’s research revealed that MSC-EVs intravenous infusion in rats with spinal cord injury exhibited significant improvements in locomotors functions compared with EV-free group (167). Moreover, MSC-derived EVs alleviated inflammatory response after spinal cord injury in mice triggering polarization of macrophages from pro-inflammatory M1 to pro-repair M2 phenotype and reducing pro-inflammatory cytokine level of TNF-α, IL-6, MCP-1, and MIP-1α while increasing anti-inflammatory cytokines: IL-4 and IL-10 in injured spinal cord tissue (166). Similarly, MSC-EVs attenuate inflammation in rat model of spinal cord injury resulting in the decrease of reactive microglia and astrocytes (168). The effect was accompanied by the expansion of suppressor cells in the blood and spleen of EV recipients.

It has been shown that MSC-EVs ameliorate liver injury as well. In lethal hepatic failure in mice, systemic administration of human MSC-EVs decreased hepatic necrosis and prolonged survival of animals (169). Similarly, BM-MSC secretome attenuated hepatocyte apoptosis and diminished liver damage induced by CCl_4_ in rats (170). MSC-EVs protected the liver from hypoxia-induced injury or from ischemia-reperfusion harm. Mice that received the injection of MSC-derived EVs before the ischemia revealed hepatocyte proliferation demonstrated by an increased number of Ki-67–positive cells and decrease of the expression of inflammation-associated genes (171). Similarly, immunosuppressive effect of MSC-EVs on liver injury animal model was reported by Tamura et al. The expression of mRNA for pro-inflammatory cytokines: IL-1, IL-2, TNF-α, IFN-γ was reduced while anti-inflammatory cytokines: TGF-β and HGF, and the number of T regulatory cells increased in the liver tissue (172).

The therapeutic efficacy of MSC-EVs for acute or chronic kidney disease has been proven experimentally. Bruno et al. proved that EVs isolated from human mesenchymal bone marrow stem cells support epithelial cell survival in acute renal failure, stimulating their proliferation and preventing apoptosis (173). In an animal model of renal ischemia/reperfusion injury, EVs have been shown to decrease epithelial/reperfusion injury. EVs have been shown to decrease epithelial tubular cell damage and increase cell proliferation and kidney function (174, 175). Some studies have shown that the renoprotective effect of EVs is caused by kidney neovascularization. Infusion of EVs improved renal capillary density and reduced kidney fibrosis in a rat model of acute kidney injury (176). Recent studies have shown that MSC-EVs also have a beneficial effect in treatment of drug-induced nephropathy inhibiting mitochondrial apoptosis in epithelial tubular cells and inducing cell proliferation (177). Moreover, in several preclinical studies of kidney injury animal models MSC-EVs therapy reduced inflammation by decreasing the presence of inflammatory cytokines: IL-1β, IL-6, and TNF-α (178–180).

The use of MSC-derived EVs has been shown to have a therapeutic effect in several experimental models of lung diseases. MSC-derived EVs infused into mice in an acute lung injury model induced by E. coli endotoxin restored protein permeability and increased fluid clearance in the alveolus (181, 182). Similarly, in a pig model of influenza virus-induced acute lung injury, intra-tracheal infusion of MSC-EVs alleviated lung lesions, inhibiting apoptosis of influenza-infected lung epithelial cells. In addition, MSC-EVs exhibited an immunomodulatory effect suppressing TNF-α and increasing IL-10 secretion in the alveolus after injury (183). MSC secretome therapy also has shown promise in chronic progressive respiratory disease which accompanies idiopathic pulmonary fibrosis. Human BM-MSC-EVs infused intravenously into bleomycin-induced pulmonary fibrotic mice restored collagen content, improved pulmonary morphology and restored lung architecture (184). Moreover, systemic administration of MSC-derived EVs modulate immune response by the decrease of inflammatory cell influx and altering alveolar macrophages toward an anti-inflammatory phenotype (184, 185). The beneficial effect of MSC-EVs injection was confirmed on *ex vivo* perfused human model of bacterial pneumonia. It was revealed that microvesicles derived from MSCs enhanced the alveolar fluid clearance and reduced lung protein permeability; additionally pretreatment of MSCs with polyinosinic:polycytidylic acid improved the antimicrobial activity of microvesicles (186).

Beneficial effects of MSC-EVs have been observed in wound and bone healing. Local multi-point injection of EVs around burn wound animal models promoted re-epithelialization, enhanced angiogenesis and accelerated wound closure (187, 188). Application of human MSC-EVs into femoral fractures in mice accelerated fracture healing (189). Similarly, repeated injections of EVs prepared from human MSCs promoted regeneration of osteochondral defects in a rat femur injury model (190). Recently, MSC-EVs combined with various scaffold materials have been shown to encourage bone repair in rodent calvarial bone defects enhancing angiogenesis and osteogenesis (191, 192).

EVs isolated from MSCs used in the model of inflammatory bowel disease induced by tri-nitrobenzene sulfonic acid injection in rats, EVs derived from rat BM-MSCs attenuated colonic inflammation *via* a markedly decrease in IL-1β and an increase in IL-10 expression (193). A similar study showed that human umbilical cord MSC-EVs reduced TNF-α, IL-1β, IL-6, iNOS, and IL-7 genes while increasing the expression of IL-10 gene in the colon tissues relieving inflammatory bowel disease’s symptoms in mice with dextran sulfate sodium induced colitis (194).

The therapeutic potential of MSC-EVs also has been proven in various animal models of organ and tissue injury including immune-related conditions i.e. graft-versus-host disease (GVHD), autoimmune diseases, sepsis etc. Adipose derived MSC-EVs infusion in a rat model of sepsis induced by cecal ligation lowered the levels of inflammatory mediators: IL-1β, TNF-α, MMP-9, NF-κB in lungs and kidneys of experimental animals (195). In murine models of acute GVHD, MSC-EVs reduced the number of cytotoxic cells and decreased the serum levels of IL-2, TNF-α and IFN-γ in peripheral blood (196, 197).

**Figure 3:**
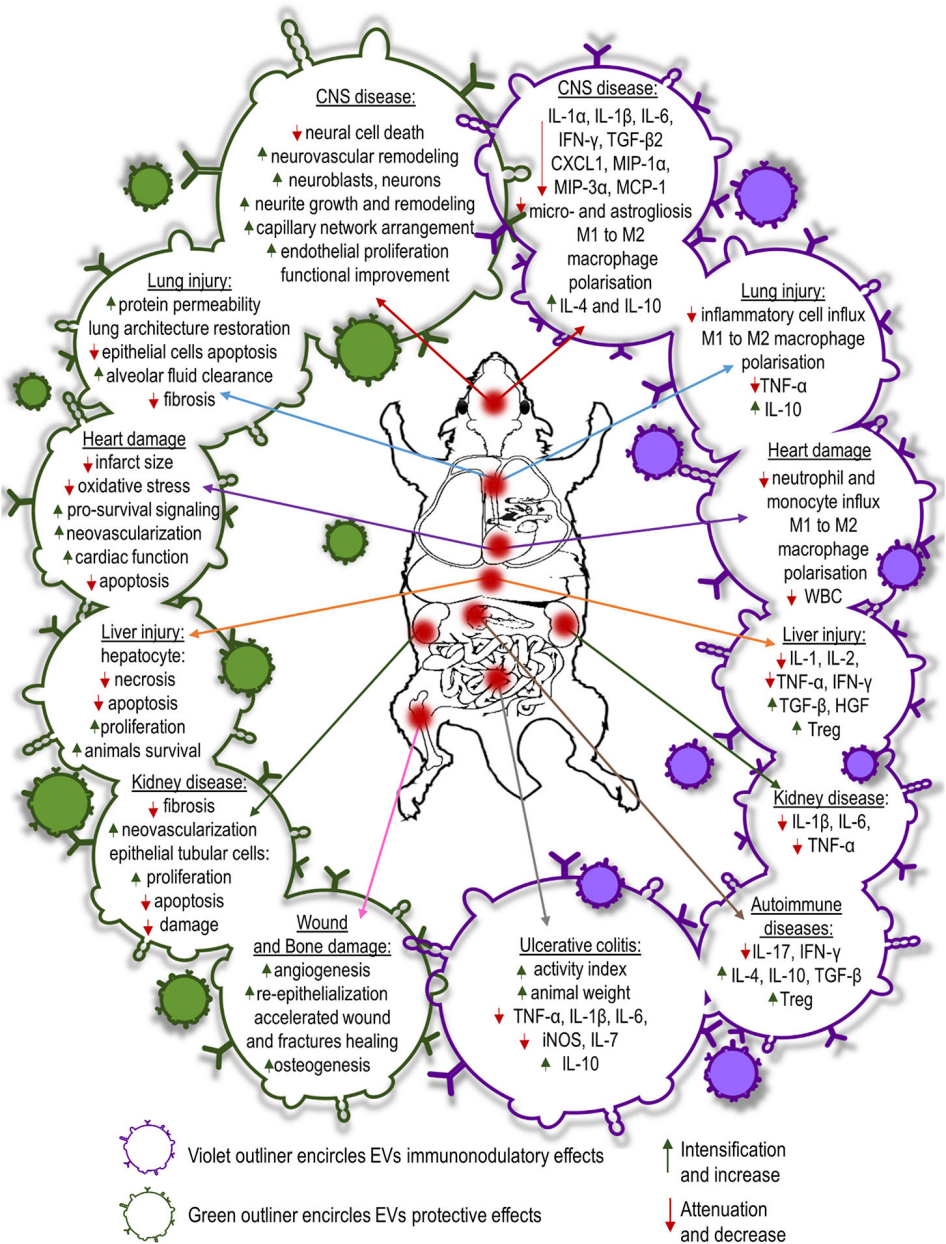
Summary of protective, regenerative or immunomodulatory capabilities of MSC-EVs administered in experimental models of different diseases.

Immunomodulatory effects of EVs have been reported in a few studies of autoimmune diseases. Bai et al. showed the ameliorative effect of local administration of MSC-derived exosomes in inhibiting migration of inflammatory cells to the retina and the significant reduction in IL-17– and IFN-γ–producing T cell subsets along with the significant increase in the CD25+Foxp3+ T regulatory cells in a rat model of experimental autoimmune uveitis (198). A recent study of MSC-EVs infusion into streptozotocin-induced of the type 1 diabetes mellitus mice reported by Nojehdehi et al. demonstrated the decrease of IL-17 and IFN-γ levels with the elevation of anti-inflammatory cytokines: IL-4, IL-10, and TGF-β in concordance with the significant increase in the Treg cell ratio in the spleen of EVs treated mice (199) (**Figure 3**).

The overview of 59 experimental studies with the administration of MSC-EVs in the different diseases models was presented in **Table 1**. The analysis revealed that the most frequently applied source of MSC-EVs was bone marrow (38 studies). At the second place MSC-EVs from umbilical cord and adipose tissue were used (10 studies and 6 studies, respectively). Less frequently other sources were applied such as: umbilical cord blood, embroynic stem cells, cardiac progenitors, myocardium, menstrual fluid, choroin, and embryo. Moreover, the analysis revealed that the most frequently used type of injected vesicles were exosomes which were examined in 29 studies. At the second place, the whole fraction of extracellular vesicles was applied, explored by 23 research groups. Microvesicles were analyzed only in 6 out of 59 studies. Importantly, only 11 studies investigated vesicles immobilized on scaffolds or derived from genetically modified cells; whereas 48 studies focused on naive, unmodified vesicles.

**Table 1:** Overview of an application of MSC-EVs in experimental studies.

Disease	MSC-EVs’ source	MSC-EVs’ type	MSC-EVs’ modification	MSC-EVs’ isolation procedure	Donor	Compatibility	Host	MSC-EVs’ dose	Administration route	Main effect	Ref.Nr
Myocardial ischemia/reperfusion injury	Embryonic stem cells	Exosomes	No	Size-exclusion fractionation on a HPLC + filtration	Human	Xeno	Mouse	0.4 μg	Intravenous	- Reduction of infarct size	(139)
Myocardial ischemia/reperfusion injury	Embryonic stem cells	Conditioned medium	No	–	Human	Xeno	Pig	2.0 mg	Intravenous, Intracardiac	-Reduction of myocardial nuclear oxidative stress -Reduction of TGF-beta signalling and apoptosis -Reduction in infarct size -Improvement of systolic and diastolic cardiac performance	(140)
Myocardial ischemia/reperfusion injury	Embryonic stem cells	Exosomes	No	Size-exclusion fractionation on a HPLC + Ffltration	Human	Xeno	Mouse	16 μg/kg 4 μg/kg 1 μg/kg	Intravenous	-Reduction of infarct size -Significant reduction of local and systemic inflammation -Restoration of bioenergetics, reduction of oxidative stress, activation of pro-survival signalling -Enhancement cardiac function and geometry	(141)
Myocardial ischemia**/**reperfusion injury	Cardiac progenitors	Exosomes	No	Filtration + precipitation	Mouse	Allo	Mouse	from 5 × 10^5^ MSCs	Intracardiac	-Inhibition of cardiomyocyte apoptosis	(142)
Dilated cardiomyopathy	Bone marrow	Exosomes	No	Differential ultracentrifugation	NQ		Mouse	300 µg	Intravenous	-Improvement of cardiac function, attenuation of cardiac dilation, cardiomyocytes apoptosis -Reduction of expression levels of inflammatory factors, decrease of the inflammatory cells - Attenuation of the pro-inflammatory macrophages	(143)
Myocardial ischemia-reperfusion injury	Umbilical cord blood	Exosomes	miRNA-181a over-expressing-exosomes	Differential ultracentrifugation + filtration	Human	Xeno	Mouse	200 μg	Intracardiac	- Immune-suppressing effect of miRNA-181a exosomes - Stronger therapeutic effect of miRNA-181a exosomes	(144)
Polymicrobial sepsis	Bone marrow	Exosomes	No	Filtration + differential ultracentrifugation	Mouse	Allo	Mouse	2 μg/g	Intravenous	- Cardioprotective effect	(129)
Myocardial ischemia/reperfusion injury	Bone marrow	Exosomes	Exo(GATA-4)	ExoQuick-TC precipitation	Rat	Allo	Rat	from 4 × 10^6^ MSCs	Intracardiac	-Restoring of cardiac contractile function and reduction of infarct size	(145)
Myocardial ischemia**/**reperfusion injury	Bone marrow	Extracellular vesicles	No	Differential ultracentrifugation	Human	Xeno	Rat	80 μg, from 2 × 10^6^ MSCs	Intracardiac	-Enhancement of blood flow recovery -Reduction of infarct size -Preservation of cardiac systolic and diastolic performance	(146)
Myocardial infarction	Myocardium	Exosomes	No	PEG precipitation	Mouse	Allo	Mouse	50 μg	Intracardiac	-Enhancement of cardiac - Increase of cardiomyocyte proliferation angiogenesis -Preservation of heart function	(147)
Myocardial infarction	Bone marrow	Exosomes	Exo(CR4)	ExoQuick-TC precipitation	Rat	Allo	Rat	NQ	Intracardiac	-Increase of angiogenesis -Reduction of infarct size -Improvement of cardiac remodeling	(148)
Myocardial infarction	Bone marrow	Exosomes	No	Differential ultracentrifugation	Human	Xeno	Mouse	4 μg	Subcutaneous	- Pro-angiogenic effects	(149)
Traumatic brain injury	Bone marrow	Secretome	No	Ultrafiltration+ heparin-agarose column	Human	Xeno	Rat	500 µg	Intravenous	-Attenuation of motor deficits and cerebral infarction -Reduction of neuronal loss and apoptosis -Increase of VEGF-positive cells in the ischemic cortex -Functional outcome improvement	(153)
Traumatic brain injury	Bone marrow	Exosomes	No	ExoQuick-TC kit	Rat	Allo	Rat	100 μg	Intravenous	-Improvement of functional recovery - Promotion of endogenous angiogenesis and neurogenesis –Reduction of inflammation	(154)
–	Menstrual fluid, chorion, bone marrow, umbilical cord	Exosomes Extracellular vesicles	No	Differential ultracentrifugation	Human	Xeno	Rat, Mouse	3 μg	–	- Growth-stimulating effects of exosomes on longest neurite in cortical neurons and neurite outgrowth in dorsal root ganglia neurons	(155)
Stroke	Bone marrow	Exosomes	No, miR-133b+ Exo	Filtration + differential ultracentrifugation	Rat	Allo	Rat	from 1 × 10^7^ MSCs	Intravenous	- Improvement of neurite remodelling -Improvement of functional recovery	(156)
Stroke	Bone marrow	Exosomes	No	Differential ultracentrifugation	Rat	Allo	Rat	–	–	- Increase of the neurite branch number and total neurite length	(157)
Transient global Ischemia	Bone marrow, adipose tissue	Extracellular vesicles	No	ExoQuick-TC kit	Mouse	Allo	Mouse	200 µg	Intracerebral	- Restoration of impaired basal synaptic transmission and synaptic plasticity –improvement of spatial learning and memory	(158)
Traumatic brain injury	Bone marrow	Extracellular vesicles	No	Scalable chromatography	Human	Xeno	Mouse	30 μg	intravenous	- suppression of neuroinflammation - rescue of pattern separation and spatial learning impairments	(159)
Traumatic brain injury	Bone marrow	Exosomes	No	ExoQuick-TC kit	Human	Xeno	Rat	100 μg 3 × 10^9^ particles	Intravenous	- Improvement of functional outcome -Promotion of endogenous angiogenesis and neurogenesis –reduction of neuroinflammation - Enhancement of spatial learning	(160)
Stroke	Bone marrow	Extracellular vesicles	No	PEG precipitation + ultracentrifugation	Human	Xeno	Mouse	from 2 × 10^6^ MSCs	Intravenous	- Improvement of neurological impairment -Long-term neuroprotection - Enhanced angioneurogenesis - Attenuation of postischemic immunosuppression in the peripheral blood	(161)
Brain injury	Bone marrow	Extracellular vesicles	No	PEG precipitation + ultracentrifugation	Human	Xeno	Rat	from 1 × 10^8^ MSCs/kg	Intraperitoneal	- Prevention of neuronal cell death -Restoration of white matter microstructure -Reduction of gliosis -Improvement of long-lasting cognitive functions	(162)
Stroke	Adipose tissue	Extracellular vesicles	No	miRCURYTM exosome Isolation kit	Rat	Allo	Rat	100 μg	Intravenous	- Improvement of functional recovery, fiber tract integrity, axonal sprouting and white matter repair markers	(163)
Focal brain injury	Bone marrow	Extracellular vesicles	No	Differential ultracentrifugation	Human	Xeno	Rat	1.3 × 10^9^ particles from 5 × 10^6^ MSCs	Intra-arterial	- Decrease of cell activation i.e., astrocytes, microglia, and infiltration of leucocytes, including T cytotoxic cells - Decrease of pro-inflammatory cytokines and chemokines	(164)
Fetal brain ischemia	Bone marrow	Extracellular vesicles	No	PEG precipitation	Human	Xeno	Sheep	from 4.0 × 10^7^ MSCs	Intravenous	- Reduction of the total number and duration of seizures - Preservation of baroreceptor reflex sensitivity - Tendency to prevent hypomyelination	(165)
Spinal cord injury	Umbilical cord	Exosomes	No	Differential ultracentrifugation	Human	Xeno	Mouse	20 μg 200 μg	Intravenous	- Improvement of functional recovery - Down-regulation of the inflammatory cytokines	(166)
Spinal cord injury	Bone marrow	Extracellular vesicles	No	Differential ultracentrifugation	Rat	Allo	Rat	40 μg from 1 × 10^6^ MSCs	Intravenous	- Reduction of brain cell death - Enhancement of neuronal survival and regeneration – Improvement of motor function	(167)
Spinal Cord Injury	Bone marrow	Extracellular vesicles	No, from MSCs stimulated with TNF-α + IFN-γ	Sequential filtration	Human	Xeno	Rat	1 × 10^9^ particles/ml	Intravenous	- Attenuation of neuroinflammation – Improvement of functional recovery	(168)
Hepatic Failure	Bone marrow	Extracellular vesicles	No	Differential ultracentrifugation	Mouse Human	Allo Xeno	Mouse	2 × 10^8^ to 2 × 10^10^ particles	Intravenous, Intraperitoneal	- Reduction of hepatic injury - Modulation of cytokine expression - Increase of survival	(169)
Liver injury	Bone marrow	Exosomes, Secretome	No	Differential ultracentrifugation	Rat	Allo	Rat	50 μg	Intravenous	- Improvement of liver regeneration	(171)
Hepatic ischemia reperfusion injury	Bone marrow	Extracellular vesicles	No	Differential ultracentrifugation + filtration	Human	Xeno	Mouse	1 × 10^9^ particles	Intravenous	-Reduction of hepatic necrosis - Increase of the amount of hepatocytes - Repression of the transcription of inflammation-associated genes - Attenuation of liver damage and improvement of organ regeneration	(171)
Liver injury	Bone marrow	Exosomes	No	Differential ultracentrifugation	Mouse	Allo	Mouse	10 μg	Intravenous	- Decreases in ALT, liver necrotic areas, and the extent of apoptosis - Increase in the cell proliferation - Enhancement of anti-inflammatory cytokines and T regulatory cells	(172)
Acute kidney injury	Bone marrow	Microvesicles	No	Differential ultracentrifugation	Human	Xeno	Mouse	15 μg	Intravenous	- Acceleration of the morphologic and functional recovery -Induction of proliferation of tubular cells	(173)
Acute and chronic kidney injury	Bone marrow	Microvesicles	No	Differential ultracentrifugation	Human	Xeno	Rat	30 μg	Intravenous	- Protection from acute kidney injury - Inhibition of apoptosis - Stimulation of tubular epithelial cell proliferation - Reduction of the impairment of renal function - Protection from later chronic kidney disease	(174)
Renal tubular cells injury	Bone marrow	Extracellular vesicles	No	Differential ultracentrifugation	Human	Allo	Human	3 × 10^9^ particles	–	- miRNAs of EVs are involved in the repair and recovery process in proximal tubular epithelial cells	(175)
Kidney ischemia reperfusion injury	Umbilical cord	Extracellular vesicles	No	Differential ultracentrifugation	Human	Xeno	Rat	100 µg from 5 × 10^5^ MSCs	Intravenous	- Reduction of cell apoptosis - Enhancement of cell proliferation - Increase of capillary vessel density -Reduction of renal fibrosis	(176)
Acute kidney injury	Umbilical cord	Exosomes	No	Differential ultracentrifugation	Human	Xeno	Rat	200 μg	Intra-renal	- Prevention from nephrotoxicity by activation of autophagy	(177)
Acute kidney injury	Bone marrow	Extracellular vesicles	No EVs with downregulation of microRNA	Differential ultracentrifugation	Human	Xeno	Mouse	2.2 × 10^8^ particles from 7.5 × 10^4^ MSCs	Intravenous	- Induction of morphologic and functional recovery by wild-type EVs not by EVs with downregulation of microRNA - microRNA depletion reduced intrinsic regenerative potential of EVs	(178)
Unilateral renovascular disease	Adipose tissue	Extracellular vesicles	No	Differential ultracentrifugation	Pig	Auto	Pig	from 10 × 10^6^ MSCs	Intra-renal	- Bearing pro-angiogenic properties restoring the renal microcirculation and in turn hemodynamics and functions	(179)
Renal ischemia-reperfusion injury	Umbilical cord	Microvesicles	No	Differential ultracentrifugation	Human	Xeno	Rat	100 μg from 5 × 10^5^ MSCs	Intravenous	- Mitigation of renal cell apoptosis - Enhancement of proliferation - Alleviation of inflammation - Suppression of the expression of chemokines and decrease the number of macrophages in the kidney - Improvement of renal function and abrogation of renal fibrosis	(180)
Acute lung injury	Bone marrow	Microvesicles	Ang-1 mRNA deficient MVs	Differential ultracentrifugation	Human	Xeno	Mouse	from 1 × 10^6^ MSCs	Intratracheal	- Deteriorative lung inflammation and a failure to restore pulmonary capillary permeability after Ang-1 mRNA deficient MVs injection	(181)
Acute lung injury	Bone marrow	Microvesicles	No	Differential ultracentrifugation	Human	Xeno	Mouse	30.9 ± 17.0 μg from 3 × 10^6^ MSCs	Intratracheal	- Reduction in pulmonary edema and lung protein permeability - Reduction in inflammation	(182)
Acute lung injury	Bone marrow	Extracellular vesicles	No	Differential ultracentrifugation	Pig	Allo	Pig	80 μg/kg	Intratracheal	- Reduction of virus shedding, influenza virus replication, production of proinflammatory cytokines - Alleviation of influenza virus-induced lung lesions	(183)
Pulmonary fibrosis	Bone marrow	Extracellular vesicles	No	Density flotation	Human	Xeno	Mouse	8.6 × 10^8^ particles from 5 × 10^6^ MSCs	Intravenous, Intracardiac	- Prevention and reversion of bleomycin-induced pulmonary fibrosis - Promotion of an immunoregulatory, anti-inflammatory monocyte phenotype	(184)
Acute respiratory distress syndrome	Bone marrow	Extracellular vesicles	EV–treated alveolar macrophages	Differential ultracentrifugation	Human	Xeno	Mouse	from 7.5 × 10^6^ cells/ml	Intranasal	- Reduction of inflammation - Amelioration of lung injury	(185)
Lung pneumonia	Bone marrow	Microvesicles	No	Differential ultracentrifugation	Human	Allo	Human	from 2 × 10^7^ or 4 × 10^7^ MSCs	Intravenous	-Increase of the alveolar fluid clearance – Reduction of the protein permeability -Lower bacterial load in the injured alveolus - Enhancement of the antimicrobial activity of MVs after pretreatment with polyinosinic:polycytidylic acid	(186)
Wound healing	Umbilical cord	Exosomes	No	Differential ultracentrifugation + filtration	Human	Xeno	Rat	200 µg	Subcutaneous	- Acceleration of re-epithelialization - Reduction of heat stress-induced apoptosis	(187)
Wound healing	Umbilical cord	Exosomes	No	Differential ultracentrifugation	Human	Xeno	Rat	200 μg	Subcutaneous	- Promotion of wound healing and angiogenesis	(188)
Fracture healing	Bone marrow	Exosomes	No	Differential ultracentrifugation	Human	Xeno	Mouse	–	Local	- Facilitation of the acceleration of fracture healing	(189)
Osteochondral defects	Embryo	Exosomes	No	HPLC fractionation	Human	Xeno	Rat	100 μg	Intra-articular	- Enhancement of gross appearance and improvement of histological scores - Complete restoration of cartilage and subchondral bone	(190)
Calvarial defects	Adipose tissue	Exosomes	Immobilized on the polydopamine-coating PLGA scaffolds	Differential ultracentrifugation + filtration	Human	Xeno	Mouse	25 μg/ml	Local	- Enhancement of bone regeneration - Osteoinductive effects and capacities of promoting MSCs migration and homing in the newly formed bone tissue	(191)
Calvarial defects	Bone marrow	Extracellular vesicles	EVs in HyStem-HP hydrogel	Differential ultracentrifugation and ultrafiltration	Human	Xeno	Rat	100 μg	Local	- More bone formation in the critical-size calvarial bone defects	(192)
Colitis	Bone marrow	Extracellular vesicles	No	Differential ultracentrifugation	Rat	Allo	Rat	50, 100, 200 μg	Intravenous	- Attenuation of the severity of colitis - Down regulation of pro-inflammatory cytokines - Modulation of anti-oxidant/oxidant balance - Moderation of the occurrence of apoptosis	(193)
Inflammatory bowel disease	Umbilical cord	Exosomes	No	Density gradient + Differential ultracentrifugation	Human	Xeno	Mouse	400 μg	Intravenous	- Relieved the severity of the disease - Increase of IL-10 – Decrease of pro-inflammatory cytokines - Decrease of the infiltration of macrophages into the colon tissues	(194)
Sepsis syndrome	Adipose tissue	Exosomes	No, apoptotic-exosomes	Differential ultracentrifugation + filtration	Rat	Allo	Rat	100 µg	Intravenous	- Improvement of survival and suppression of the inflammatory reactions by healthy exosomes superior to apoptotic exosomes	(195)
Graft-versus-host disease	Bone marrow	Exosomes	No	Differential ultracentrifugation + filtration	Human	Xeno	Mouse	100 μg	Intravenous	- Prolongation of the survival and diminishment of the clinical and pathological scores – amelioration of the fibrosis in the skin, lung, and liver - Reduction of activation and infiltration into lungs of CD4+ T cells, inhibition of IL-17-T cells and induction of IL-10- regulatory cells, reduction of pro-inflammatory cytokines	(196)
Graft-versus-host disease	Umbilical cord	Extracellular vesicles	No	Differential ultracentrifugation	Human	Xeno	Mouse	200 μg	Intravenous	- Alleviation of the manifestations of aGVHD and reduction of the mortality - Lower absolute numbers of CD3+CD8+ T cells; reduction serum levels of pro-inflammatory cytokines; a higher ratio of CD3+CD4+ and CD3+CD8+ T cells; higher serum levels of IL-10	(197)
Autoimmune uveitis	Umbilical cord	Exosomes	No	Differential ultracentrifugation + filtration	Human	Xeno	Rat	10, 20, 50, 100 μg	Periocular	- Reduction of the intensity of ongoing EAU by reducing the infiltration of T cell subsets, and other inflammatory cells into the eyes	(198)
Autoimmune diabetes	Adipose tissue	Exosomes	No	Differential ultracentrifugation	Mouse	Auto	Mouse	50 µg	Intraperitoneal	- Increase of the levels of anti-inflammatory cytokines, decrease in the levels of pro-inflammatory cytokines - Increase in the T regulatory cell ratio - increase in islets - Stability in body weight, blood glucose levels in a survival	(199)

The above mentioned studies showed that in the most studies human MSC-EVs were applied (40 studies). Subsequently, rat MSC-EVs (10 studies), mouse MSC-EVs (7 studies) and swine MSC-EVs (2 studies) were administrated. The effect of transplanted MSC-EVs was most commonly explored in mouse and rat experimental models (28 and 26 researches, respectively). Rarely, the human, ovine and swine models were investigated (2, 1 and 3 studies, respectively). Regarding the compatibility of MSC-EVs’ injection into the different models, the most frequently used was xenogenic transplantation, applied in 38 of presented studies, showing no adverse effects. This confirms the safety of application of MSC-EVs derived from the different donors to the host without requiring immunological compatibility. Then, 19 allogenic and 2 autologous administrations were used in the referred studies.

Up to now, the most frequently used method for MSC-EVs’ isolation was differential ultracentrifugation utilized in 42 referred investigation. In many research studies, this technique was combined with other methods such as: filtration, precipitation or density gradient separation. Other commonly used techniques were: precipitation or its combination with the other techniques (7 studies), filtration or its combination with other methods (6 studies) or application of exosome isolation kit (6 studies). The isolated MSC-EVs were assessed according to the measurement of the level of proteins (39 studies), the number of particles (8 studies) or the number of cells from which EVs derived (18 studies). Moreover, the analysis showed that the most frequently used administration route of EVs was intravenous injection, applied in 32 studies. Alternatively, MSC-EVs were administered using many other routes such as: intracardiac (eight studies), intraperitoneal (three studies), subcuntaneous (three studies), intratracheal (three studies), local injection (three studies), or intra-renal infusion (two studies). Finally, intracerebral, intra-arterial, intranasal, intra-articular, and periocular route of EVs’ administration were applied in individual cases of referred studies.

### MSC-EVs in Clinical Trials

Inspired by promising results of therapeutic effect of MSC-EVs observed in preclinical studies, the first clinical trials using MSC-EVs were established. Currently, over 100 clinical studies involving exosomes are listed in www.clinicaltrials.gov. The majority of these trials focus on the use of endogenous exosomes for diagnostic purposes. However, the number of clinical applications of MSC-EVs is rather scanty (200, 201).

The first case report of application MSC-EVs was published by Kordelas et al. in 2014. Individual treatment with the exosomes isolated from the supernatant of allogeneic BM-MSCs has been applied to therapy-refractory GVHD patient. Four doses of MSC-EVs obtained from the supernatant of 4 × 10^7^ BM-MSCs were administered every 2 to 3 days. Clinical symptoms improved significantly shortly after treatment. The cutaneous and mucosal GVHD showed remarkably response after 2 weeks and it was stable for several months following MSC-EVs infusion. MSC-EVs therapy allowed to reducing the dosage of steroids from 125 mg/d to 30 mg/d. Also, the reduced pro-inflammatory cytokine response of the patient PBMN was observed following the third dose of MSC-EVs (202).

Katagiri et al. published the first clinical report of alveolar bone regeneration with the secretome derived from allogeneic BM-MSCs (Lonza). The clinical trial was undertaken to evaluate the safety and therapeutic effect of MSC-CM in patients with severe bone resorption due to periodontitis who need bone augmentation prior to dental implants. Conditioned medium from BM-MSCs soaked to collagen sponge was implanted at the laterally maxillary sinus wall in 8 patients. No local or systemic complications were reported. Radiographic evaluation followed by histological assessment performed 6 months later revealed early bone formation in all cases. Furthermore, infiltration of inflammatory cells was observed less often in these bone specimens (203).

MSC-EVs have also been studied in patients with chronic kidney disease (CKD). The trial was conducted by Nassar et al. to assess the safety and therapeutic efficacy of umbilical cord MSC-EVs in ameliorating the progress of chronic kidney disease. Forty CKD patients were enrolled in phase II/III clinical studies. The patients have been divided into two groups, the first group (20 CKD patients) was treated by two doses of MSC-EVs, the first dose injected intravenous (IV) and the second dose administrated intra-renal arteries (IA) one week later. The control group (20 CKD patients) received saline solution infused IV. The therapy was safe, there were any significant adverse effects noticed after the treatment up to one year of studies. Infusion of MSC-EVs ameliorated clinical symptoms and transiently (3–6 months) improved the kidney function, reducing blood urea and serum creatinine while increasing eGRP. Moreover, CKD patients treated with MSC-EVs demonstrated amended inflammatory immune reaction observed by the significant increase of TGF-β1 and IL-10 and the decrease in TNF-α level in plasma (204).

To sum up, MSC-EVs seem to be interesting approach to be studied in clinical trials of different diseases especially those accompanied with inflammatory component. There are currently several trials evaluating MSC-EVs reported to Clinicaltrials.gov. Most of them are in recruiting or not yet recruiting status (**Table 2**). The beneficial effects of MSC-EVs could be enhanced by bioengineering and genetic modifications, stimulation with different growth factors or drug encapsulation. However, there are challenges to optimize MSC-EVs clinical use (205, 206). Further research is warranted to establish optimal culture conditions of MSCs, suitable therapeutic doses and administration routs but also the standardized protocols for EVs isolation and storage.

**Table 2:** Clinical trials using MSC-EVs therapy in different disorders.

Study title	Condition of disease	Intervention treatment	Trial Phase	Status	Trial ID
A tolerance clinical study on aerosol inhalation of mesenchymal stem cells exosomes in healthy volunteers	Healthy	Aerosol inhalation of allogeneic AD MSC-EVs (2 × 10^8^ or 4 × 10^8^ or 8 × 10^8^ or 12 × 10^8^ or 16 × 10^8^ or 20 × 10^8^)	Phase I	Recruiting	NCT 04313647
A safety study of IV stem cell-derived extracellular vesicles (UNEX-42) in preterm neonates at high risk for BPD	Bronchopulmonary dysplasia	IV infusion of BM-MSC-EVs (20 or 60 or 200 pmol phospholipids/kg body weight)	Phase I	Recruiting	NCT 03857841
A clinical study of mesenchymal stem cell exosomes nebulizer for the treatment of ARDS	Acute Respiratory distress syndrome	Aerosol inhalation of allogeneic MSC-EVs (2 × 10^8^ or 8 × 10^8^ or 16 × 10^8^ particles for 7 days)	Phase I	Recruiting	NCT 04602104
A clinical study of allogeneic human adipose-derived mesenchymal progenitor cell exosomes (haMPC-Exos) nebulizer for the treatment of carbapenem-resistant gram-negative bacilli-induced pulmonary infection	Pulmonary infection caused by gram-negative bacilli resistant to carbapenems	Aerosol inhalation of MSC-EVs (8 × 10^8^ or 16 × 10^8^ nanovesicles for 7 days)	Phase I/II	Recruiting	NCT 04544215
A pilot clinical study on inhalation of mesenchymal stem cells exosomes treating severe novel coronavirus pneumonia	COVID-19	Aerosol inhalation of allogeneic MSC-EVs (2 × 10^8^ nanovesicles/3 ml for 5 days)	Phase I	Recruiting completed	NCT 04276987
Allogenic mesenchymal stem cell derived exosome in patients with acute ischemic stroke	Acute ischemic stroke	Stereotactic intra-parenchymal infusion of allogeneic MSC-EVs enriched by miR-124	Phase I/II	Recruiting	NCT 03384433
The safety and the efficacy evaluation of allogenic adipose MSC-exos in patients With Alzheimer’s disease	Alzheimer Disease	Allogeneic MSC-EVs administered by nasal drip (5 µg or 10 µg or 20 µg in 1 ml twice a week for 12 weeks)	Phase I/II	Recruiting	NCT 04388982
Comparative effectiveness of arthroscopy and non-arthroscopy using mesenchymal stem cell therapy (MSCs) and conditioned medium for osteoarthritis	Osteoarthritis, knee	Intraarticular injection of UC-MSC-EVs (2 cc/knee and 2 cc/knee 4 weeks later)	Phase I/II	Recruiting	NCT 04314661
Effects of ASC secretome on human osteochondral explants	Osteoarthritis	ASC-Esc or ASc conditioned medium applied ex vivo on osteochondral explants	Phase I	Not-yet recruiting	NCT 04223622
MSC Evs in dystrophic epidermolysis bullosa	Dystrophic epidermolysis bullosa	Allogeneic MSC-EVs applicate locally on lesion once a day for 60 days	N/A	Not-yet recruiting	NCT 04173650
MSC-exos promote healing of MHs	Idiopathic macular hole	UC-MSC-Exosomes (20 µg or 50 µg in 10 µl of PBS dripped into vitreous cavity around MH)	Early Phase I	Recruiting	NCT 003437759
Effect of UMSCs derived exosomes on dry eye in patients with cGVHD	Dry eye symptoms in patients with chronic GVH	Artificial tears applicate for 2 weeks followed by UMSC-exosomes (10 µg/drop four times a day for 14 days)	Phase I/II	Recruiting	NCT 04213248
Exosome of mesenchymal stem cells for multiple organ dysfunction syndrome after surgical repair of acute type A aortic dissection	Multiple organ dysfunction syndrome (MODS)	Intravenous infusions of MSC-EVs (150 mg once a day for 14 days)	N/A	Not-yet recruiting	NCT 04356300
Effect of microvesicles (MVs) and exosomes therapy on β-cell mass in type I Diabetes Mellitus	Diabetes mellitus Type 1	Two intravenous infusions of UC-MSC -MVs. The first close of UC-MSC-exosomes in a dose sups from 1.2 to 1.5 × 10^6^/UC-MSC/kg, the second dose after 7 days of UC-MSC-vesicles in a dose of sups from 1.2 to 1.5 × 10^6^ UC-MSCs/kg	Phase II/III	Unknown	NCT 02138331
iExosomes in treating participants with metastatic pancreas cancer with KrasG12D mutation	Metastatic pancreatic ductal adenocarcinoma harboring Kras G12D mutations	Intravenous injection of MSC-exosomes with KRASG 12D siRNA over 15–20 min on days: 1, 4, and 10. Treatment repeats every 14 days for up to 3 courses	N/A	Not-yet recruiting	NCT 03608631

*Information obtained from https://clinicaltrials.gov on 2^nd^ December 2020.

### Therapeutic Potential of MSCs and MSC-EVs in COVID-19

The outbreak of pneumonia in Wuhan, China in the late 2019 led to the detection of a new contagion - severe acute respiratory syndrome coronavirus 2 (SARS-CoV-2), causing coronavirus disease 2019 (COVID-19) (207). The virus has rapidly spread worldwide and has become a global threat unheard of for decades (208). The considerable variation of clinical course of infected people from nearly 50% being asymptomatic but contagious to 10% of severe cases and 1% to 2% of deaths makes the disease difficult to trace. More importantly, the disease causes the massive burden on the healthcare systems due to rapidly appearing new serious cases. It was early shown that the severity of the disease results not only from the lytic activity of the virus, but also from the dysregulation of the immune system and the abnormal immune response in the form of cytokine storm (209). Moreover, pulmonary fibrotic consequences may follow COVID-19 (210).

Numerous preclinical studies have demonstrated the immunomodulatory and antifibrotic properties of MSCs (211–213). Their clinical safety in treating bacterial sepsis and the ability to temporarily reduce the value of anti-inflammatory cytokines in the system have been already confirmed (214, 215). The first case series of patients with COVID-19 indicated the possibility of obtaining therapeutic benefits from the application of MSCs into patients with severe COVID-19 (215). It spurred numerous clinical trials across the globe (216) and recommendations for their compassionate use (217). The safety and encouraging tolerance of MSCs by COVID-19 patients was shown afterward (218). The randomized clinical trial performed in Nanjing, China demonstrated positive effects of the intravenous administration of human umbilical cord blood MSCs in patients with severe COVID-19 (219).

It has also been proposed that many therapeutic activities of MSCs are executed through extracellular vesicles (EVs) released by them (105). Therefore, it was anticipated the anti-COVID-19 action of EVs (220, 221). A case series of 24 patients revealed no adverse effects and suggested the possibility for blood oxygenation restoration, cytokine storm downregulation, and immunity reconstitution after intravenous infusion of EVs (222).

In summary, MSCs and EVs derived from them might be an attractive option for treating severe and critically ill COVID-19 patients. This therapeutic strategy exploits the immunomodulatory and antifibrotic characteristics of MSCs and their derivatives. There are reported highly promising early phase clinical trials with these investigational products; though large, randomized, and double-blinded clinical trials are still needed for an approval by regulatory bodies and their widespread application.

## Concluding Remarks and Future Perspectives

A growing body of evidence supports the notion that MSC-derived EVs have similar immunomodulatory properties as their parental cells. Administration of MSC-EVs in different experimental settings and clinical trials of various diseases, in particular those with an inflammatory component, promote tissue regeneration and healing after the injury, while attenuating inflammation. Alternative therapeutic strategies involving EVs avoid risks associated with the use of stem or genetically modified cells. Moreover, replacing cell transplantation with the use of EVs isolated from them can simplify the application and reduce the risk of micro embolism during systemic cell infusion. However, there is still a discrepancy between the different isolation protocols as a result of which is obtained the various subsets of EVs, non-vesicular particles and protein aggregates’ contamination. It is also a big challenge to obtain a large-scale EVs’ production (223). Moreover, a choice of an appropriate strategy to isolate well-purified EVs is crucial to remain EVs-related functions and achieve the desired therapeutic effect (224). Therefore, there is a pivotal need to find a consensus on gold-standard method in order to MSC-EVs application in clinical studies. Thus, MSC-EVs offer promising novel therapeutics, however standardization and production of EVs need to be established before they are approved for routine use in the clinic.

## Author Contributions

SD, MJ, and BL invented the initial conception of the paper. SD and BL wrote the manuscript. AA designed the figures. SD, AA, MJ, BL edited the manuscript and approved the final version of the manuscript. All authors contributed to the article and approved the submitted version.

## Funding

This work was supported by NCR&D grant EXPLORE ME within the “STRATEGMED I” program. SD was supported by START 2020 scholarship sponsored by The Foundation for Polish Science.

## Conflict of Interest

The authors declare that the research was conducted in the absence of any commercial or financial relationships that could be construed as a potential conflict of interest.

## Abbreviations

BDNF, brain-derived neurotrophic factor; bFGF, basic fibroblast growth factor; CKD, chronic kidney disease; COVID-19, coronavirus disease 2019; COX-2, cyclooxygenase-2; DCs, dendritic cells; EGF, epidermal growth factor; EV(s), extracellular vesicle(s); FGF, fibroblast growth factor; GDNF, gliogenic neurotrophic factor; GM-CSF, granulocyte and macrophage colony-stimulating factor; G-CSF, granulocyte colony stimulating factor; GVHD, graft-versus-host disease; hBM-MSCs, human bone marrow stem cells; HGF, hepatocyte growth factor; IDO, indolamine 2,3-dioxygenase; IGF-1, insulin-like growth factor; IFN-γ, interferon-γ; LIF, leukemia inhibitory factor; MHC, major histocompatibility complex; MI/R, myocardial/reperfusion; miRNA, microRNA; mRNA, messenger RNA; MSC-EVs, EVs derived from MSCs; MSC(s), mesenchymal stem cell(s); NGFβ, beta nerve growth factor; NK, natural killer; PDGF, platelet-derived growth factor; PGE-2, prostaglandin E-2; SARS-CoV-2, severe acute respiratory syndrome coronavirus 2; SCF, stem cell growth factor; SDF-1, stromal-derived factor 1; TBI, traumatic brain injury; TGFβ, transforming growth factor β; Th, T helper; Treg, T regulatory; VEGF, vascular endothelial growth factor.
